# Proportion of patients with *Demodex* blepharitis in ophthalmology clinics in Europe: the Eos study

**DOI:** 10.1038/s41433-025-04130-4

**Published:** 2025-12-05

**Authors:** Mayank A. Nanavaty, Oliver Findl, Francesco Carones, Sheraz M. Daya, Gerd Geerling, Thomas Kohnen, Emil D. Kurniawan, David Lockington, Erik L. Mertens, Mario Nubile, Radhika Rampat, Filomena Ribeiro, Aida Hajjar Sesé, David Shahnazaryan, Elizabeth Yeu, H. Burkhard Dick

**Affiliations:** 1 University Hospitals Sussex NHS Foundation Trust, Worthing, UK; 2 Brighton & Sussex Medical School, Brighton, UK; 3 Hanusch Hospital, Vienna, Austria; 4 ADVALIA Vision, Milan, Italy; 5 Centre for Sight, London, UK; 6 University Hospital Düsseldorf, Düsseldorf, Germany; 7 Dept. of Ophthalmology, Goethe University Frankfurt, Frankfurt, Germany; 8 Royal Free Hospital NHS Foundation Trust, London, UK; 9 Tennent Institute of Ophthalmology, Gartnavel General Hospital, Glasgow, UK; 10 Medipolis Eye Center, Antwerp, Belgium; 11 University “G. d’Annunzio” of Chieti and Pescara, Chieti, Italy; 12 Royal Free London Hospital NHS Foundation Trust, London, UK; 13 Hospital da Luz and University of Lisbon, Lisbon, Portugal; 14 King’s College Hospital NHS Foundation Trust London/ Queen Mary’s Hospital, London, UK; 15 Virginia Eye Consultants & CMO Tarsus, Norfolk, USA; 16 University Eye Clinic Bochum, Bochum, Germany

**Keywords:** Infectious diseases, Microbiology

Blepharitis, an inflammatory condition of the eyelid margins, is caused and driven by various factors, including the overgrowth of *Demodex* mites [1]. *Demodex* is frequently present in the sebaceous glands of the skin at non-pathologic levels in the general population but is not routinely detected in clinical practice. Failure to identify and treat the infestation in patients with *Demodex* blepharitis (DB) can result in chronic, progressive, or refractory disease.

Patients with DB frequently experience bothersome symptoms, such as dryness, fluctuating vision and eyelid itching, with significant negative impact on daily activities and overall well-being [2]. DB is associated with contact lens intolerance and can worsen other ocular conditions, contribute to meibomian gland dysfunction (MGD) and complicate outcomes of intraocular surgery. Without effective treatment, DB and MGD can also contribute to serious complications, including punctate keratitis, corneal melting and infection [3, 4].

DB is diagnosed through clinical examination, with the presence of sleeve collarettes, solidified excretions that form a cylindrical collar around the base of the eyelash follicle, serving as a pathognomonic sign [1]. Collarettes can be readily identified by inspecting the upper lid lash line when a patient looks down during a standard slit-lamp examination, and confirms infestation without the need for lash epilation or mite counting [5]. Despite the potential serious downstream effects, patients may go undiagnosed or misdiagnosed for years, in part due to substantial overlap of symptoms with dry eye disease and other ocular surface or lid margin disorders, as well as underappreciation of the condition and a general lack of awareness among eye care professionals to look for collarettes actively [4, 6].

In the absence of an approved prescription therapy in Europe, DB remains challenging to treat. Current options have limited efficacy and include routine eyelid hygiene, tea tree oil wipes and other products, topical and oral antibiotics, topical anti-inflammatory agents, microblepharoexfoliation and intense pulsed light therapy.

The prevalence of DB has been reported in various studies worldwide. More than half of patients attending eye clinics in the US and Japan exhibit signs of DB. Two recent US studies have reported *Demodex* infestation rates of 55% and 58% among patients presenting for eye examinations for varying reasons [4, 7], while 66% of patients visiting clinics in Japan exhibited DB [8]. European literature is more limited. Studies from individual ophthalmology clinics have found flakes or collarettes in 45% of those presenting with ocular discomfort, and collarettes in 58% of patients attending for any reason [9, 10].

In this study (Eos), cohorts of approximately 50 consecutive patients attending ophthalmology clinics for any reason, with no exclusions, were screened for DB. Patients were checked for the presence of collarettes by asking them to look down during slit-lamp examination (at ×10 or ×16 magnification), with each eyelid assessed separately. The presence of more than 10 collarettes per eyelid was also recorded. This work was a non-interventional observational audit and did not require formal ethical approval in accordance with applicable UK and EU regulations.

**Fig. 1:**
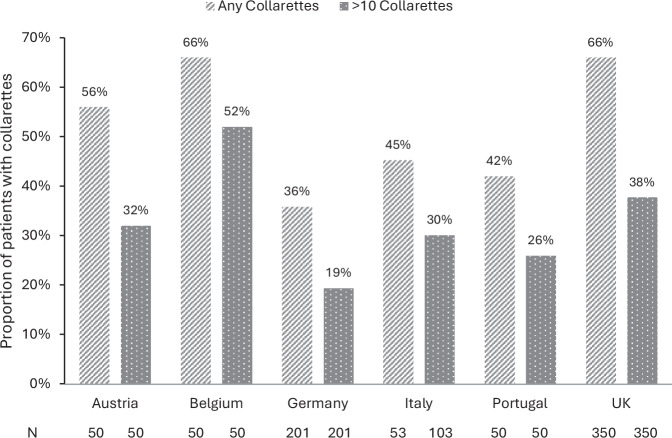
Proportion of patients with *Demodex* blepharitis by country. The presence of any collarettes was reported in 1of 2 cohorts from Italy; therefore, the total number of patients with any collarettes is derived from one cohort only (53 patients), whereas the proportion of patients with >10 collarettes is derived from both cohorts (103 patients).

**Fig. 2:**
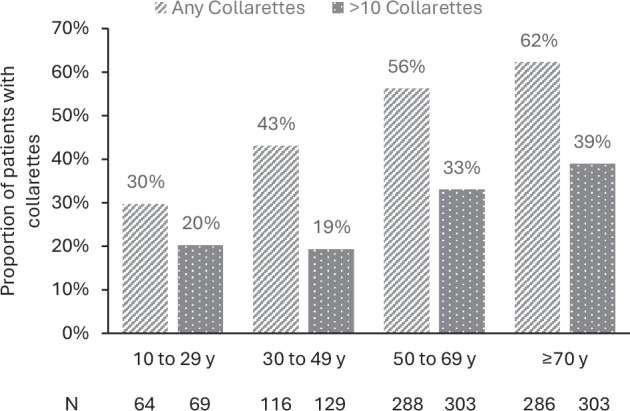
Proportion of patients with *Demodex* blepharitis by age. y, years; the presence of any collarettes was reported in 1of 2 cohorts from Italy; therefore, the proportion of patients with any collarettes for each age group is derived from a subset of the population (754 patients), whereas the proportion of patients with >10 collarettes is derived from the whole sample (*N* = 804).

A total of 804 patients attending clinics in Austria, Belgium, Germany, Italy, Portugal, and the UK were included in the analysis of >10 collarettes, with a median age of 64 years (IQR 50–75). Additionally, 754 patients were also checked for the presence of any collarettes (data for one cohort were not collected). Collarettes were recorded in 54% of patients (95% confidence interval [CI] 51–58%; 409/754), and a significant proportion (32%; 95% CI 31–38%; 257/804) had a more severe infestation (>10 collarettes per eyelid). While sample sizes in individual countries are not large enough to draw conclusions, we noted that the proportion of patients with DB detected in clinic visits varied by country, with the highest proportion reported by ophthalmologists in the UK and Belgium (66%) and the lowest by ophthalmologists in Germany (36%; Fig. 1). It has long been established that the prevalence of DB increases with age. In our study, DB was detected in 30% of patients under 20 years of age, increasing to 62% in those 70 years of age or older (Fig. 2). While the proportion reported in the younger group is consistent with that reported in university-based populations, other studies have reported universal presence in those aged above 70 years [1].

This study reports the frequency of DB across a broad range of ophthalmology patients. Given that collarettes are clearly visible during a slit-lamp examination when the patient looks down, the high rate of occurrence found in this study represents a valuable but often missed diagnostic opportunity, since many patients with chronic irritation might have an undiagnosed *Demodex* infestation.

The emerging clinical view is that any presence of collarettes with symptomatic blepharitis is indicative of DB and should be treated, and that patients with more than 10 collarettes should be treated even in the absence of symptoms [5, 11]. Our data support the use of collarette count as a practical and non-invasive clinical marker of DB, with more than 10 collarettes per lid potentially serving as a clinically relevant threshold to prompt treatment consideration.

This study has some limitations. Given the relatively small cohorts screened in each clinic and the inclusion of patients attending for any reason, heterogeneity in the sampled populations is likely. Variation in collarette occurrence between countries may reflect differences in demographics, case mix or occupation of the patients included, as well as differences in examination practices and environmental factors. These findings underscore the importance of standardising diagnostic criteria and increasing awareness of *Demodex*-related signs across clinical settings. The association between collarettes, symptoms and outcomes were not evaluated in this study and further research is warranted to validate and understand this patient population.

With an emerging targeted therapy for DB, early and accurate diagnosis has become increasingly actionable in this frequently overlooked and untreated disease. These findings strengthen the case for screening for the presence of collarettes, which may be particularly beneficial in individuals with chronic symptoms, concurrent MGD, contact lens intolerance and those scheduled for ocular surgery.
